# Cecal appendicitis as a rare manifestation of paracoccidioidomycosis: A case report and systematic review of the literature

**DOI:** 10.1590/1678-9199-JVATITD-2025-0015

**Published:** 2025-12-08

**Authors:** Isadora de Lima Xavier Andrade, Bruna Abdul Ahad Saad, Alexandre Albuquerque Bertucci, Marcel Arakaki Asato, João Paulo Gregório Machado, Maína de Oliveira Nunes, Eliana da Costa Alvarenga de Brito, James Venturini, Sandra Maria do Valle Leone de Oliveira, Cláudia Elizabeth Volpe-Chaves, Anamaria Mello Miranda Paniago

**Affiliations:** 1 Maria Aparecida Pedrossian University Hospital, Campo Grande, MS, Brazil.; 2 Regional Hospital of Mato Grosso do Sul, Campo Grande, MS, Brazil.; 3 Federal University of Mato Grosso do Sul, Campo Grande, MS, Brazil.; 4 Oswaldo Cruz Foundation (Fiocruz), Campo Grande, MS, Brazil.

**Keywords:** Paracoccidioidomycosis, Paracoccidioides brasiliensis, Appendicitis, Appendectomy, Acute abdomen

## Abstract

**Background:**

Paracoccidioidomycosis (PCM) is a systemic mycosis endemic to Latin America, with the acute/subacute form predominantly affecting children and young adults. Cases of cecal appendicitis caused by *Paracoccidioides* spp. have rarely been reported. This study aimed to describe the clinical manifestations and evolution of a case of cecal appendicitis due to PCM and to conduct a systematic literature review.

**Case presentation:**

We present the case of a 20-year-old male with generalized lymphadenopathy who was diagnosed with PCM and treated with oral trimethoprim-sulfamethoxazole. After the initial improvement, the patient returned with clinical deterioration. The treatment was changed to liposomal amphotericin B. Six days later; the patient developed an acute abdomen and underwent exploratory laparotomy with appendectomy. Histopathological examination confirmed acute granulomatous appendicitis due to PCM, and the patient showed postoperative clinical improvement. A systematic review were conducted using Embase, Web of Science, Lilacs, Medline, LIEPCS, PubMed, SciELO, and Gray Literature databases. Of the ten identified articles included in the systematic review, most case reports with a low risk of bias were found in South American countries. Seven patients were confirmed appendicitis due to PCM through biopsy, whereas one had confirmed PCM at another site. Two patients were initially misdiagnosed with Crohn's. Most studies have reported favorable outcomes.

**Conclusion:**

Appendicitis caused by PCM is rare, even in endemic countries. It has a benign course when properly treated with both clinical and surgical management. This should be considered in the differential diagnosis of acute abdomen with lymphadenopathy in endemic regions.

## Background

Paracoccidioidomycosis (PCM) is a systemic mycosis endemic to Brazil, accounting for more than 80% of reported cases of the disease [1]. PCM is also found in almost all Latin American countries [2]. The etiological agents belong to the genus *Paracoccidioides*, with the main representatives being the *P. brasiliensis* complex (including *P. brasiliensis* sensu stricto, *P. americana*, *P. restrepiensis*, and *P. venezuelensis*) and *P. lutzii* [3].

These fungi are found in nature and human infections occur through the inhalation of infectious propagules and spores. The main risk factor is soil exposure, usually during the first two decades of life, which is the period during which infection is acquired. However, clinical manifestations of the disease often appear several years later [4].

PCM manifests in two main clinical forms: the chronic form, which accounts for most cases, and the acute/subacute form (ASF). The chronic form is characterized by symptoms that persist for more than six months and predominantly affect the lungs, upper airways, and digestive tract. ASF is more common in children, adolescents, and young adults and mainly involves the organs of the mononuclear phagocytic system, including the lymph nodes, bone marrow, liver, and spleen [5].

Involvement of the cecal appendix in PCM, which is part of the gut-associated lymphoid tissue (GALT), has rarely been described in the literature, and its diagnosis can be challenging. Therefore, the present study aimed to describe the clinical, diagnostic, and therapeutic manifestations of appendicitis caused by *Paracoccidioides* spp. through a case report and systematic literature review.

## Case presentation

The CARE guidelines [6] were used in this case report. The patient provided informed consent for publication of the case, and the project was approved by the Institutional Review Board of the Federal University of Mato Grosso do Sul, Brazil (protocol number 2.102.875). Written informed consent for publication was obtained from the patient. 

### Case report

A 20-year-old male patient, born and residing in Campo Grande, MS, working for an urban waste selective collection company, presented with a history of a nine-kilogram weight loss and generalized lymphadenopathy for three months, initially affecting the cervical region and later involving the bilateral axillary and inguinal regions. One week before admission, he developed febrile peaks of 38°C, fatigue, odynophagia, and hyporexia. The patient denied any comorbidities, medication use, previous hospitalizations or surgeries, or history of allergies.

On physical examination, lymphadenopathy was observed bilaterally in the preauricular, retroauricular, right occipital, superficial and deep cervical, tonsillar, subclavicular, submental, axillary, and inguinal lymph node chains, with sizes ranging from one to two centimeters (cm), painful, and fixed. Larger lymph nodes were found in the left superficial cervical, left subclavicular, submental, left retroauricular, and right superficial cervical chains, varying between three and seven cm, and were painful and fixed. Laboratory tests revealed normocytic and normochromic anemia, leukocytosis with a left shift, elevated C-reactive protein (CRP), and hypoalbuminemia (see Additional file 1). 

**Figure 1.**
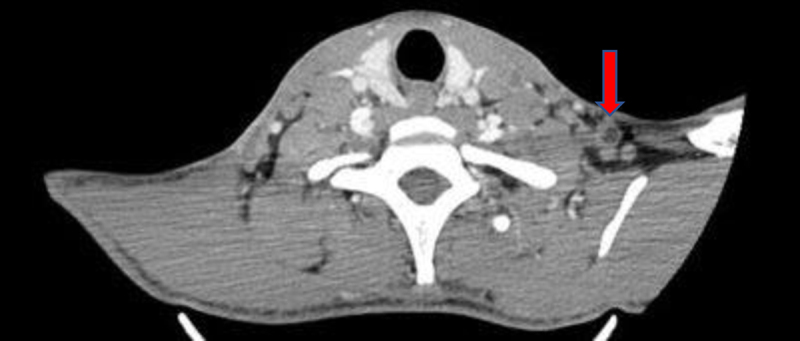
Cervical region tomography highlighting a necrotic lymph node (red arrow).

The antibody test for anti-*Paracoccidioides* using double immunodiffusion in agar gel was positive, with a titer of 1:64. Computed tomography (CT) of the cervical region revealed enlarged lymph nodes in all the cervical, supraclavicular, and axillary lymph node chains, several of which exhibited liquefied necrotic centers (Figure 1). No serological tests for other fungi were performed. 

The chest CT showed no abnormalities. Abdominal CT scan showed no abnormalities in the liver, spleen, kidneys, aorta, inferior vena cava, bladder, or prostate. The pancreas was normally positioned but was located near the densified mesenteric fat, which limited the evaluation of its contours. There were adenopathies in the inguinal chains with lymph nodes measuring up to 0.42 cm in the short axis, thickened and hyperechoic mesenteric fat, and adjacent intestinal loops exhibiting inflammatory features suggestive of diffuse enteropathy. 

Histological examination of the inguinal lymph nodes revealed chronic granulomatous lymphadenitis with numerous refringent, rounded structures of varying sizes within the histiocytes. Direct mycological examination revealed structures suggestive of *Paracoccidioides* spp. (+++), which were confirmed by culture. 

Treatment with trimethoprim-sulfamethoxazole (TMP-SMX) was initiated orally at a dose of 800/160 mg every 8 h, and the patient was discharged with clinical improvement after four days of treatment. Itraconazole was not initiated because of intestinal involvement and erratic absorption. After 11 days, the patient returned to the outpatient clinic with poor adherence to pharmacological treatment, fever, and persistent diffuse lymphadenopathy. An attempt was made to use intravenous fluconazole; however, the patient did not tolerate it and was maintained on TMP-SMX, taking two tablets orally (PO) every 8 h.

**Figure 2.**
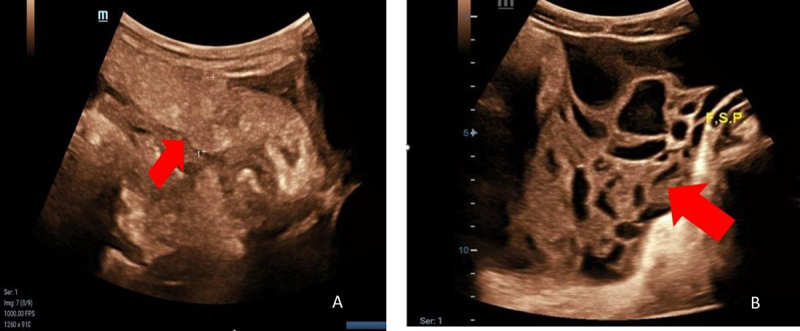
Abdominal ultrasound highlighting acute inflammatory changes suggestive of cecal appendicitis. **(A)** Acute cecal appendicitis. **(B)** Fluid collection with thin septations.

The patient returned to the outpatient clinic after three weeks and presented with significant abdominal pain and increased cervical lymphadenopathy. Liposomal amphotericin B (L-AmB) was administered at a dose of 3 mg/kg/day in a day hospital setting. Six days after starting L-AmB, the patient experienced worsening abdominal pain, particularly in the right inguinal region, with a negative Blumberg sign but positive Dunphy and Rovsing signs. An abdominal ultrasound revealed a fluid collection with thin septations in the posterior cul-de-sac, measuring 8.6 × 5.3 cm, and an elongated structure with ill-defined borders in the right iliac fossa, measuring 8.0 × 2.6 cm, suggestive of acute cecal appendicitis (ACA) (Figure 2). Given the compatible clinical presentation, no additional abdominal CT was performed, and the patient underwent an appendectomy.

**Figure 3.**
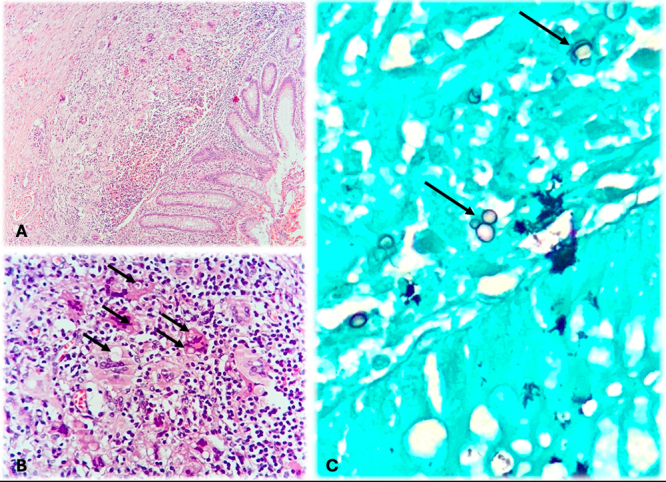
Histopathological examination of cecal appendicitis caused by paracoccidioidomycosis. **(A)** Histopathological examination showing an intense granulomatous inflammatory process in the cecal appendix wall (40x). **(B)** Fungal structures and inflammatory infiltrate with multinucleated giant cells (400x, arrows indicating the fungal structures). **(C)** Grocott-Gomori special staining showing numerous typical structures of *Paracoccidioides* sp. (400x).

Histopathological examination of the cecal appendix showed an intense granulomatous inflammatory process in the wall of the appendix with multinucleated giant cells and numerous fungal structures typical of *Paracoccidioides* spp., highlighted by Grocott-Gomori staining (Figure 3).

The patient was discharged from the hospital with progressive clinical improvement and continued treatment with TMP- SMX. The patient remains under outpatient follow-up.

The timeline of the reported case is described in Additional file 2.

### Systematic review

A systematic review was conducted, as recommended by the AMSTAR 2 tool (A MeaSuremen Tool to Assess Systematic Reviews) [7]. The description was based on the Preferred Reporting Items for Systematic Reviews and Meta-Analyses (PRISMA) [8]. The systematic review protocol was registered under number CRD42024508229 on PROSPERO (International Prospective Register of Systematic Reviews): https://www.crd.york.ac.uk/prospero/.

#### Search strategies and eligibility criteria

The research question in the protocol was as follows: What is the clinical course of patients with cecal appendix involvement by *Paracoccidioides* spp.? Patients with a confirmed diagnosis of cecal appendicitis caused by *Paracoccidioides* spp., with an assessment of clinical manifestations, treatment, and outcomes, were included.

EPPI Reviewer 6 software (version 6.15) was used (https://eppi.ioe.ac.uk/cms/Default.aspx?tabid=2914).

Two reviewers (ILXA and BAAS) screened titles, abstracts, and descriptors to identify articles for analysis. A third reviewer (CEVC) screened all excluded abstracts and any conflicts were resolved.

Searches were conducted in August 2023, and the search strategy was updated until July 2024. The Embase, Web of Science, LILACS, MEDLINE, LIEPCS, PubMed, and SciELO databases were used. Searches for unpublished studies were conducted in Open Grey (http://www.opengrey.eu/), the CAPES Thesis and Dissertation Bank, the Brazilian Digital Library of Theses and Dissertations (BDTD), Open Access Theses and Dissertations (OATD), MedNar, and WorldWidescience.org. The complete search strategy is shown in Additional file 3.

To construct search strategies for each database, descriptors from MeSH (Medical Subject Headings) and other related terms, including EMTREE terms, were selected. The search strategy consisted of the following terms: (((appendicitis[MeSH Terms]) OR (appendices, omental[MeSH Terms])) OR (small intestine[MeSH Terms])) AND ((((paracoccidioides brasilienses[MeSH Terms])) OR (paracoccidioidomycoses[MeSH Terms]))) and “paracocc*” and “apendic*.”

Articles that were irrelevant to the theme or were duplicates were excluded. Articles meeting the inclusion criteria were read in full and the data obtained from each study were recorded by the EPPI Reviewer. Only articles that met the inclusion criteria for clinical cases of PCM involving the cecal appendix were selected for data extraction.

After reading the full texts, articles that did not meet the inclusion criteria were excluded and the reasons for exclusion were recorded.

The search results are reported in the Preferred Reporting Items for Systematic Reviews and Meta-Analyses (2020) flowchart and an evidence map. Articles included for full reading that could not be accessed after several attempts were excluded. The authors of these articles were contacted via email or the ResearchGate platform (https://www.researchgate.net/), and only one author responded to our inquiry. Some articles could not be found because they were old.

#### Data extraction

Data were extracted by two reviewers (IXLA and BAAS), and the accuracy and completeness were verified by a third reviewer (CEVC). A table was used to characterize the articles based on the following information: study type, age, sex, clinical presentation, diagnostic method, pharmacological treatment, and outcomes.

#### Risk of bias assessment

The risk of bias assessment was carried out by one reviewer (CEVC) using the guidelines recommended by the Joanna Briggs Institute (JBI) [9]. Full verification of all judgments was performed by a second reviewer (BAAS). The JBI critical appraisal checklist for textual evidence includes the following options: N (no), N/A (not applicable), U (unclear), and Y (yes). The risk of bias was interpreted based on the proportion of items met: low risk of bias (most critical items were met), moderate risk of bias (some important items were not met), and high risk of bias (many critical items were not met).

#### Evidence map

To characterize the volume, characteristics, outcomes, and quality of the available studies, we developed an evidence map to visually summarize the main information found in the systematic review, using the EPPI Reviewer 6 software (version 6.15) (https://eppi.ioe.ac.uk/cms/Default.aspx?tabid=2914). 

## Results

Using this search strategy, 91 articles based on case reports were identified. The articles were written in Portuguese, English and Spanish, with the publication years ranging from 1913 to 2023. Twelve articles were excluded because of duplication, and 74 articles were eligible for title and abstract reading.

**Figure 4.**
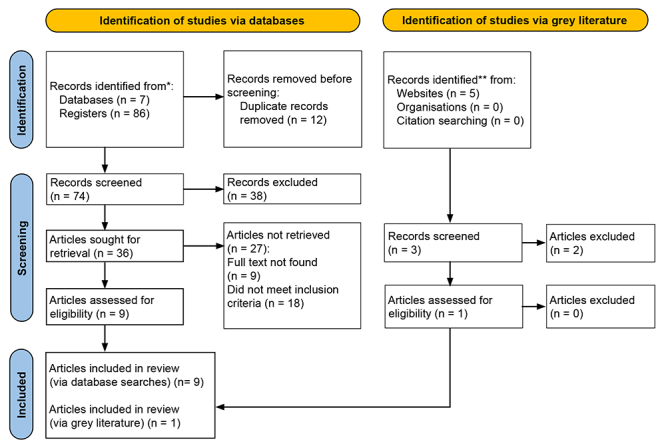
Flowchart of study selection. PRISMA 2020 flow diagram showing included searches of databases and grey literature. *Embase, Web of Science, Lilacs, Medline, LIEPCS, PubMed, and SciELO. **Grey Literature: thesis and dissertation database (CAPES), Digital Library of Theses and Dissertations (BDTD), open access theses and dissertations (OATD), WorldWidescience and MedNar. Source: Page et al. [8]. For more information, visit: http://www.prisma‐statement.org/.

Thirty-six articles were eligible for full-text review. Twenty-seven articles were excluded after reading their full texts. The results of the search strategy are shown in Figure 4.

Ten articles [10-19] met the inclusion criteria for clinical cases of PCM affecting the cecal appendix, and the characteristics of the studies are described in Table 1. We also included the data from our case reports (Table 1). A meta-analysis was not conducted due to the small number of included articles.

**Table 1.** Characterization of included studies with cecal appendicitis caused by paracoccidioidomycosis.

Reference	Country	Age (years)/Gender	PCM presentation	Pulmonary symptoms (chest X-ray/CT)	Gastrointestinal symptoms	Other sites	Diagnostic method (Was there an appendix perforation?)	Pharmacological treatment	Death
Vianna [10]	Brazil	32/ M	NR (Probable acute/subacute form)	Yes (NR)	Dyspnea, weight loss, diffuse lesions throughout the body, abdominal pain, and diarrhea	Skin, lymph nodes, mucosa, subcutaneous tissue (gummas), adrenal glands, lungs, spleen, liver, kidneys, CNS	HP of ACA and other organs (No appendix perforation)	No	Yes
César et al. [11]*	Brazil	NR/ NR*	NR	NR	NR	Skin	NR	NR	NR
Barbosa et al. [12]	Brazil	26 M	NR	NR	Sudden abdominal pain with nausea, vomiting, and sweating	Lymph nodes	HP of ACA (No appendix perforation)	NR	NR
Bittencourt et al. [13]	Brazil	4 M	Acute/subacute form	Yes (Chest X-ray showing interstitial infiltrate at the bases)	Fever, dry cough, weight loss, dyspnea, and cervical lymph nodes. Hepatosplenomegaly and abdominal mass	Lymph nodes, spleen, liver	HP of ACA and cervical lymph node. Culture positive for *P. brasiliensis* (No appendix perforation)	Did not receive pharmacological treatment	Yes
Navas et al. [14]**	Venezuela	20 NR	Acute/subacute form	No (NR)	NR	Lymph nodes, some presenting a scrofulous appearance	NR (PCM serology was negative)	TMP-SMX	No
Muñoz Urribarri et al. [15]	Peru	5 M	Acute/subacute form	No (NR)	Fever, vomiting, abdominal distension, and abdominal pain	Isolated abdominal lymph nodes	HP of ACA PCM serology was negative (No appendix perforation)	Itraconazole 10 mg/kg/day PO	No
Gava et al. [16]	Brazil	20 M	NR	No (NR)	Hematochezia, enterorrhagia, and weight loss	Lymph nodes, skin, and mucosa	HP of oral lesion and abdominal CT with description of ACA (No appendix perforation)	L-AmB IV (No surgery)	No
Luna-Vilchez et al. [17]	Peru	10 M	Acute/subacute form	No (NR)	Fever, generalized lymphadenopathy, weight loss, diarrhea, pain in the right iliac fossa	Lymph nodes, liver, and spleen	HP of ACA (No appendix perforation)	L-AmB 3mg/kg/day IV for 14 days	No
Sales et al.*** [18]	Brazil	33 M	NR	No (NR)	Periumbilical and lower abdomen pain associated with nausea, hyporexia, and weight loss	No	HP of ACA (No appendix perforation)	Antifungal treatment was administered but not reported	No
Marinho Falcão et al. [19]	Brazil	42 M	Acute/subacute form	No (NR)	Odynophagia, weight loss, disseminated skin lesions, diffuse lymphadenopathy and abdominal pain	Skin and mucous membranes, lymph nodes, adrenal gland	HP of skin lesions and ACA; direct examination; serology reactive for PCM (1:256) (No appendix perforation)	Itraconazole 400 mg/day PO for 24 months	No
Present study ****	Brazil	20 M	Acute/subacute form	No (Normal chest CT)	Weight loss, disseminated lymphadenopathy, abdominal pain	Lymph nodes	HP of ACA serology reactive for PCM (1:64) and positive direct examination (No appendix perforation)	TMP- SMX 800/160 mg PO every 8 hours. Followed by L-AmB 3 mg/kg/day for 10 days, and then TMP- SMX PO	No

ACA, acute cecal appendicitis; CT, computed tomography; HP, histopathological; IV, intravenous; L-AmB, liposomal amphotericin B; M, male; NR, no report; PCM, paracoccidioidomycosis; PO, oral administration; TMP-SMX, trimethoprim-sulfamethoxazole

*The article refers to only one case of appendicitis in 1955. **Article not found and data extracted from the abstract. ***Data extracted from a conference poster. ****Data from this case report.

Of the 10 articles included as cases of cecal appendicitis due to PCM, one was found through a manual search.

The studies that met the criteria for the systematic review were all case reports, including one described in a thesis from 1913 [10], one study presented as a letter to the editor [16], and one presented as conference posters [18]. Seven reports were from Brazil [10-13, 16, 18, 19], two from Peru [15, 17], and one from Venezuela [14], covering the period from 1913 to 2023. Including our article, nine patients were male and two were of unspecified sex. Their ages ranged from 4-42 years.

The main symptoms were abdominal pain, weight loss, fever, and presence of lymph nodes swelling. The patients were previously healthy [10, 15-19] but two had been treated for Crohn’s disease prior to the diagnosis of PCM, indicating a misdiagnosis and had used immunosuppressants [16] and corticosteroids [19], which intensified the progression of PCM. One patient who died had a presumptive diagnosis of non-Hodgkin lymphoma due to an abdominal mass and did not present with abdominal pain [13].

Most patients did not present with any pulmonary symptoms. The majority had involvement of other sites in addition to intestinal compromise; however, in two cases [15, 18], the signs and symptoms were exclusively gastrointestinal.

The majority of cases were confirmed through histopathology of the appendix, identifying *Paracoccidioides* spp. [10, 12, 13, 15, 17-19]. One study histopathologically confirmed PCM at another site, and ACA was diagnosed using abdominal CT [16]. In two cases, the diagnosis was made postmortem [10, 13]. There have been no reports of appendiceal perforation based on histopathological findings.

In the cases described in the systematic review, two patients were treated with L-AmB [16, 17]. Two patients were treated only with itraconazole from the diagnosis [15, 19] and one received itraconazole after induction therapy with L-AmB [17]. One patient was treated with TMP-SMX [14]. One study did not report outpatient treatment following the use of L-AmB [16] and four studies did not report any pharmacological treatment instituted [10-12, 18].

Six patients showed clinical improvement after treatment [14-19]. One patient showed clinical improvement with pharmacological treatment alone and did not require surgery [16]. One patient died without receiving treatment [13]. Four articles did not report the clinical outcome [10-12, 14].

In the risk of bias assessment (JBI) for the cases included in the systematic review, five articles were rated as having a low risk of bias [10, 13, 15, 17, 19]; two reports were rated as having a moderate risk of bias [12, 16] and three articles were rated as having a high risk of bias [11, 14, 18] due to not being accessible for full-text reading (see Additional file 4). We did not exclude articles with a high risk of bias owing to the small number of reported cases. 

In Additional file 5, we present an evidence map to characterize the volume, characteristics, outcomes, and quality of the available studies. The complete evidence map can be accessed at https://eventos.matogrossodosul.fiocruz.br/mapas/apendicite_paracoc.html

## Discussion

This study reports a rare form of ACA caused by *P. brasiliensis* in a case with acute/subacute PCM (AS-PCM) following an exploratory laparotomy of the acute abdomen during the initial phase of treatment.

AS-PCM accounts for a minority of reported cases in the literature (5-10%) and primarily affects children and young adults [4] consistent with our patient's presentation, who exhibited classic signs and symptoms at onset, such as fever, weight loss, anorexia, and generalized lymphadenopathy.

Although the patient was not directly involved in rural activities, a significant epidemiological factor in PCM [20], he worked at a waste collection company and was occasionally exposed to inhaled soil particles while passing through unpaved streets, which may have been a route of infection for the fungus.

In Campo Grande, the patients’ place of origin, PCM cases were monitored at the Systemic Mycoses Outpatient Clinic of Maria Aparecida Pedrossian University Hospital. A study conducted at this reference service found that AS-PCM was more frequent among patients in their second and third decades of life, with an average age of 22.2 years and a male-to-female ratio of 3:1. The main clinical manifestations include involvement of the mononuclear phagocyte system, primarily lymphadenopathy (95.4%), hepatomegaly (40%), and splenomegaly (23.1%) [21]. Another study published in 2014 showed that in Mato Grosso do Sul, the prevalence of the ASF during the 2000-2009 decade decreased compared to the two previous decades, probably due to an intense campaign against child labor in agriculture in the country [22]. 

Our patient presented with typical features of appendicitis, including right lower quadrant pain and positive Dunphy and Rovsing signs. Laboratory findings revealed leukocytosis with left shift and elevated CRP levels. Preoperatively distinguishing between bacterial ACA and appendicitis caused by PCM is challenging and is often achieved only through histopathological examination of the resected appendix. Although appendectomy is a common and often necessary intervention for appendicitis related to PCM, follow-up with appropriate antifungal treatments is crucial to prevent recurrence or complications.

The systematic review identified only 10 documented cases of cecal appendicitis caused by *Paracoccidioides* spp. [10-19], indicating that this is a rare disease restricted to South American countries. Most of the described cases had favorable outcomes following diagnosis and treatment. One case was untreated, and the diagnosis was made only late, resulting in the patient’s death [13]. The first report of cecal appendicitis caused by PCM described in 1914 [10] was diagnosed during an autopsy. In addition, we believe that many cases are either undiagnosed or unreported. This also reflects the difficulty in identifying cases using our search strategy, necessitating the use of grey literature and references from the included studies.

The acute form of PCM was predominant in our review, and the involvement of multiple sites beyond the gastrointestinal tract was also common. Nevertheless, two reports exclusively presented gastrointestinal signs and symptoms. As Brazil is endemic for PCM, it should be considered in individuals with appendicitis who do not have other organ involvement.

In our review, we observed reports of older patients [10, 11, 12]. One case was described in a letter to the editor [16], one in a conference poster [18], and one for which we only had access to the abstract [14].

With characteristics similar to those of our case report, studies on cecal appendicitis have involved young patients between the ages of 4 and 42 years, all of whom were male and previously healthy. The main symptoms included abdominal pain, fever, weight loss, and lymphadenopathy, highlighting the presence of a systemic disease.

On the initial abdominal CT scan, the patient presented with adenopathy in the inguinal chains and thickened and hyperechoic mesenteric fat with an inflammatory appearance suggestive of diffuse enteropathy. There are numerous reports in the literature of PCM mimicking Crohn's disease or ulcerative colitis, as in the case described in 1979, due to its tendency to affect intestinal lymph nodes and cause enteropathy [23]. Furthermore, this diagnostic confusion led to inadequate treatment and consequently greater immunosuppression in the two cases reported in our review, resulting in clinical progression and surgical complications [16, 19].

Although primary infection through ingestion or gastric transmission of *Paracoccidioides* has been suggested in some studies [5, 24], this is not currently recognized as a valid mechanism of infection. The prevailing consensus is that infection occurs via inhalation of fungal propagules present in the soil, leading to a primary pulmonary infection, followed by hematogenous or lymphatic dissemination to other organs, including the gastrointestinal tract [20, 25]. The main alterations in the intestinal tract are usually found in the small and large intestines, particularly in segments rich in lymphoid tissue such as the ileum, cecum, appendix, and ascending colon [5, 24].

We opted not to initiate pharmacological treatment with itraconazole due to intestinal involvement, as absorption of this medication would likely be impaired in this case [26]. The patient was discharged on TMP-SMX 800/160 mg every 8 h, as indicated in the latest PCM consensus, which includes this as one of the treatment options [20]. In our systematic review, we observed no preference for treatment; however, in general, conventional or lipid formulations of amphotericin B may have been initially chosen in two studies [16, 17] due to the severity of the cases and difficulty with oral absorption. However, other patients were treated with oral drugs such as TMP-SMX [14], or itraconazole [15, 19]. What we observed that the most important factor was immediate initiation of treatment. The only patient who did not receive treatment progressed to death [13].

With the worsening of the clinical picture and significant abdominal pain, the possibility of a paradoxical reaction was raised, which can be explained by clinical deterioration during appropriate treatment of the infectious agent. Given the patient’s irregular medication use, the primary hypothesis was disease progression in the acute/subacute phase; however, we did not dismiss the possibility of appendicitis due to a paradoxical reaction, as an exacerbation of the immune response may have occurred after the treatment began [27].

A common characteristic of the cases in the systematic review, including the one presented in this study, was that eight of the reported cases had a diagnosis confirmed by histopathological examination of the appendix, with cases subsequently leading to appendectomy. One report highlighted the need for early diagnosis, including inflammation of the appendix caused by PCM, as in this case [16], the patient received only pharmacological treatment and did not require surgical intervention. Appendicitis likely developed because of the involvement of the intestinal lymph nodes by the fungus, leading to an ileocecal mass that caused obstruction and subsequent suppuration of the appendix [16].

In our case, histopathological analysis showed no signs of appendiceal perforation, consistent with all studies in our systematic review. Intestinal perforation is a rare complication of PCM and has been reported in only one of the fully reviewed cases. Although appendicitis was initially suspected, the diagnosis was ruled out after identifying an ileal perforation (5). In cases with early diagnosis, conservative management with antifungal therapy may be a feasible treatment option before developing complications. 

Overall, despite treatment initiation, the reported lethality for all forms of PCM was between 6.1% and 7.6% [21, 28]. In our case, the evolution was satisfactory and comparable to most cases involving cecal appendicitis reported in our review. In a study of 46 clinical cases of intestinal PCM, 12.8% of patients died [5]. In our systematic review, one patient did not present with abdominal pain suggestive of appendicitis and was initially managed as a suspected case of non-Hodgkin lymphoma, with clinical deterioration. The diagnosis of PCM with necrosis of the appendix was confirmed only after death [13]. Furthermore, the first reported case of cecal appendicitis caused by PCM was described in an autopsy study [10].

The main limitations of this study, which can also be observed in the evidence map (Additional file 5), are: 1) limited number of reported cases in the literature, primarily from South America; 2) very old studies; 3) difficulty contacting authors for article retrieval: 4) many articles were excluded because of a lack of access; 5) data were not available for some studies with a moderate and high risk of bias. The literature does not describe the diagnostic challenges in differentiating cecal appendicitis caused by PCM from other infectious or inflammatory etiologies or clinical studies addressing the best therapeutic management in such cases. Additionally, the absence of compulsory notification for the disease in Brazil hampers our ability to identify the true impact and complications of these two clinical presentations.

Satisfactorily, our patient was discharged and is currently under outpatient follow-up.

## Conclusion

Cecal appendicitis due to PCM is a rare presentation of the ASF of PCM. It affects young individuals and can be difficult to diagnose even in endemic regions of Latin America. With accurate diagnosis and appropriate treatment, including surgical intervention, outcomes can be satisfactory. Particularly in endemic areas, gastrointestinal involvement leading to cecal appendicitis due to invasive fungal disease should be considered as it may be confused with other inflammatory bowel diseases and treated with immunosuppressive therapies that can trigger dissemination and potentially unfavorable outcomes. 

### Abbreviations

ACA, acute cecal appendicitis; AmB, amphotericin B; ASF, acute/subacute form; AS-PCM, acute/subacute PCM; CRP, C-reactive protein; CT, computed tomography; D-AmB, amphotericin B deoxycholate; DM, direct mycology; HP, histopathological; L-AmB, liposomal amphotericin B; M, male; NR, no report; PCM, paracoccidioidomycosis; PO, oral administration; TMP-SMX, trimethoprim-sulfamethoxazole; US, ultrasound.

## Supplementary material

The following online material is available for this article:

Additional file 1. Laboratory test results in the diagnosis of paracoccidioidomycosis upon hospital admission.

Additional file 2. Timeline of the case report of cecal appendicitis due to paracoccidioidomycosis.

Additional file 3. Characteristics of the search strategies in electronic databases.

Additional file 4. Risk of bias of the studies included in the systematic review.

Additional file 5. Evidence map of case reports of cecal appendicitis caused by paracoccidioidomycosis.

## Availability of data and materials

 All data generated or analyzed during this study are included in this article.
