# Supplementary material for: Cecal appendicitis as a rare manifestation of paracoccidioidomycosis: A case report and systematic review of the literature
Source: J Venom Anim Toxins Incl Trop Dis. 2025 Dec 8;31:e20250015. doi: 10.1590/1678-9199-JVATITD-2025-0015 (PMC12705073; doi:10.1590/1678-9199-JVATITD-2025-0015)
Supplement: Additional file 1. [file 1678-9199-jvatitd-31-e20250015-s1.pdf]

J Venom Anim Toxins incl Trop Dis
https://doi.org/10.1590/1678-9199-JVATITD-2025-0015

Supplementary Material to "Cecal appendicitis as a rare manifestation of paracoccidioidomycosis: a case report and systematic review of the literature"

Additional file 1. Laboratory test results in the diagnosis of paracoccidioidomycosis upon hospital admission.

Test	Result	Range
**Hematimetric Index**		
Hemoglobin	10·3 g/dL	13·5–17 g/dL
Hematocrit	30·3%	41–53%
MCV	83 fL	82–97 fL
**Leucocytes series**	mm^3	mm^3/%
Leukocytes	17490	4500–10000
Neutrophil	81%	41–77%
Band neutrophil	18%	5–11%
Segmented	63%	36–66%
Lymphocyte	9%	22–44%
Monocyte	6%	2–8%
Platelets	426000	150000–450000/mm^3
**Serum parameters**		
Sodium, mEq/L	132	136–145 mEq/L
Potassium, mmol/L	6·8	3·5–5·5 mmol/L
Creatinine, mg/dL	0·6	0·5–1·2 mg/dL
Glucose, mg/dL	0	70–99 mg/dL
Urea, mg/dL	17·3	10–50 mg/dL
Magnesium, mg/dL	1·96	1·7–2·6 mg/dL
AST, U/L	22	40 U/L
ALT, U/L	21	41 U/L
Albumin, mg/dL	2·06	3·4–4·8 g/dL
C reactive protein, mg/L	173·8	0–5 mg/L
Globulin, mg/dL	4·3	3·5–5·5 g/dL
Latic dehydrogenase (LDH), U/L	233	135–225 U/L
**Serological markers**		
Treponemal test	NRS	NRS
Anti-HIV	NRS	NRS
HbsAG	NRS	NRS
Anti-HBc	NRS	NRS
Anti-HBs	NRS	NRS
Anti-HCV	NRS	NRS
CMV-IgG	NRS	NRS
CMV-IGM	NRS	NRS
Toxoplasmosis IgG	NRS	NRS
Toxoplasmosis IgM	NRS	NRS

Test	Result	Range
Rapid teste for *Leishmaniasis*	NRS	NRS
Anti-*Paracoccidioides* (DID)	1:64	

ALT, alanine aminotransferase; anti-HBc, antibody against the core antigen; anti-HBs, antibody against hepatitis B virus; AST, aspartate aminotransferase; CMV, cytomegalovirus; DID, double immunodiffusion in agar gel; HBsAg, hepatitis B surface antigen; HCV, hepatitis C virus; HIV, human immunodeficiency virus; LDH, lactate dehydrogenase; MCV, mean corpuscular volume; NRS, nonreactive serum.
