# Supplementary material for: Cecal appendicitis as a rare manifestation of paracoccidioidomycosis: A case report and systematic review of the literature
Source: J Venom Anim Toxins Incl Trop Dis. 2025 Dec 8;31:e20250015. doi: 10.1590/1678-9199-JVATITD-2025-0015 (PMC12705073; doi:10.1590/1678-9199-JVATITD-2025-0015)
Supplement: Additional file 2. [file 1678-9199-jvatitd-31-e20250015-s2.pdf]

J Venom Anim Toxins incl Trop Dis
https://doi.org/10.1590/1678-9199-JVATITD-2025-0015

Supplementary Material to "Cecal appendicitis as a rare manifestation of paracoccidioidomycosis: a case report and systematic review of the literature"

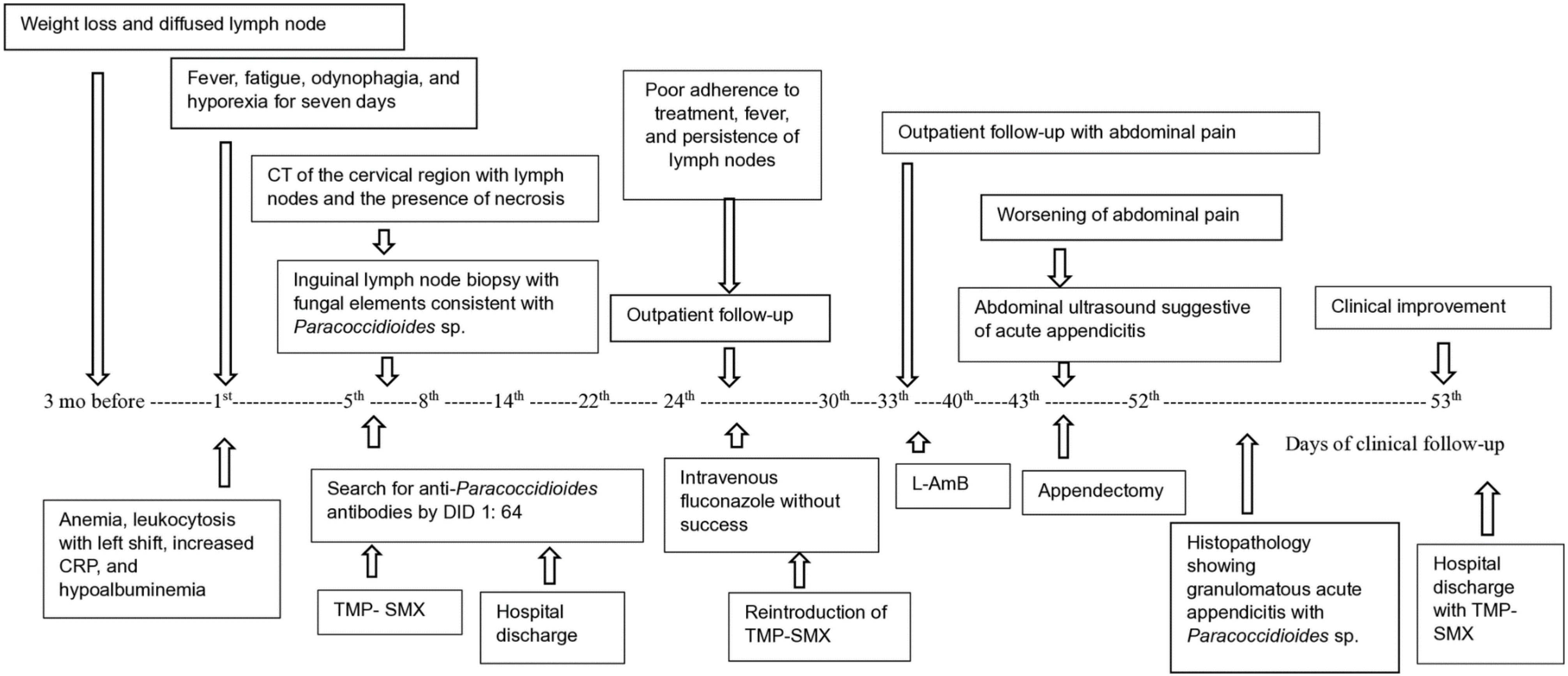

Additional file 2. Timeline of the case report of cecal appendicitis due to paracoccidioidomycosis.
CT, computed tomography; TMP-SMX, trimethoprim-sulfamethoxazole; CRP, C-reactive protein; L-AmB, liposomal amphotericin B; DID, immunodiffusion in agar gel; 3 mo, 3 months.
