# Supplementary material for: Cecal appendicitis as a rare manifestation of paracoccidioidomycosis: A case report and systematic review of the literature
Source: J Venom Anim Toxins Incl Trop Dis. 2025 Dec 8;31:e20250015. doi: 10.1590/1678-9199-JVATITD-2025-0015 (PMC12705073; doi:10.1590/1678-9199-JVATITD-2025-0015)
Supplement: Additional file 3. [file 1678-9199-jvatitd-31-e20250015-s3.pdf]

*J Venom Anim Toxins incl Trop Dis*
https://doi.org/10.1590/1678-9199-JVATITD-2025-0015

# Supplementary Material to "Cecal appendicitis as a rare manifestation of paracoccidioidomycosis: a case report and systematic review of the literature"

**Additional file 3.** Characteristics of the search strategies in electronic databases.

Database: EMBASE/MEDLINE and Preprints Address: www.embase.com/ https://bvsms.saude.gov.br/minibanners/medline/ https://www.preprints.org/
Search Date: June 8, 2024
Period covered by the search: 1946- 2024 There were no language restrictions

| Search | Strategy | Result |
|---|---|---|
| 1 | intestine AND paracoccidioides AND brasilienses OR paracoccidioidomycoses | 12 |

Database: Web of science
Address: www.web of science.com
Date: June 8, 2024
Period covered by the search: 1946- 2024
There were no language restrictions

| **Search** | **Strategy** | **Result** |
|---|---|---|
| 1 | Sources Embase, MEDLINE, Preprints<br>Query('appendicitis'/exp OR 'appendicitis' OR (appendices AND omental)) AND 'south American blastomycosis'<br>Mapped terms "appendicitis" mapped to 'appendicitis', term is exploded | 5 |

Database: PubMed
Address: www.pubmed.org
Search Date: June 8, 2024
Period covered by the search: 1946- 2024
There were no language restrictions

| **Search** | **Strategy** | **Result** |
|---|---|---|
| 1 | (((appendicitis[MeSH Terms]) OR (appendices, omental[MeSH Terms])) OR (small intestine[MeSH Terms])) AND ((((paracoccidioides brasilienses[MeSH Terms]) ) OR (paracoccidioidomycoses[MeSH Terms]))) | 9 |
| 2 | (appendicitis OR appendic*) AND paracocc* | 28 |

Database: BVS
Address: https://bvsalud.org/
Search Date: June 8, 2024, 2024
Period covered by the search: 1946- 2024
There were no language restrictions

| **Search** | **Strategy** | **Result** |
|---|---|---|
| 1 | (appendicitis OR appendices OR small intestine) AND (paracoccis OR paracoccidioidomicose) | 18 |

Search in the Grey Literature

| | | | | |
|---|---|---|---|---|
| July 20, 2024 | Banco de teses e dissertações (CAPES) | Paracocc* intestino<br>Paracocc* apêndice<br>paracocc* apendicite<br>paracocc* infecção | 0 | No found |
| July 20, 2024 | BDTD | Paracocc* intestino<br>Paracocc* apêndice<br>paracocc* apendicite<br>paracocc* infecção | 0 | No found |
| July 20, 2024 | OATD | paracocc* AND disease | 0 | No found |

| | | | | |
|---|---|---|---|---|
| | | paracocc* AND intestinal<br>paracocc* AND gastrinteritis<br>paracocc* AND appendicitis | | |
| July 20, 2024 | MedNar | paracocc* AND appendicitis<br>paracocc* appendicitis* | 0 | No found |
| July 20, 2024 | WorldWidescience.org | paracocc* and appendicitis | 1 | Marinho Falcão EM, da Costa Medeiros M, Freitas ADÁ, de Almeida Soares JC, Fernandes Pimentel MI, Quintella LP, Saraiva Freitas DF, de Macedo PM, do Valle ACF. Acute paracoccidioidomycosis worsened by immunosuppressive therapy due to a misdiagnosis of Crohn's disease. PLoS Negl Trop Dis. 2023 Jan 10;17(1):e0011023. doi: 10.1371/journal.pntd.0011023. PMID: 36626374; PMCID: PMC9870162. |
| July 20, 2024 | WorldWidescience.org | paracocc* and appendicitis | 1 | Felipe Giron, Marco Vanegas, Lina M. Rodriguez, Valentina Hernandez-Santamaria, Carlos Eduardo Rey Chaves, Jairo Ortega,<br>Intestinal Paracoccidioidomycosis: Case report and literature review,<br>International Journal of Surgery Case Reports,<br>Volume 91,<br>2022,<br>106801,<br>ISSN 2210-2612,<br>https://doi.org/10.1016/j.ijscr.2022.106801. |
| July 20, 2024 | WorldWidescience.org | paracocc* and appendicitis | 1 | Goldani, L. Z. (2011). Gastrointestinal Paracoccidioidomycosis. Journal of Clinical Gastroenterology, 45(2), 87–91. doi:10.1097/mcg.0b013e3181fae |
