# Supplementary material for: Cecal appendicitis as a rare manifestation of paracoccidioidomycosis: A case report and systematic review of the literature
Source: J Venom Anim Toxins Incl Trop Dis. 2025 Dec 8;31:e20250015. doi: 10.1590/1678-9199-JVATITD-2025-0015 (PMC12705073; doi:10.1590/1678-9199-JVATITD-2025-0015)
Supplement: Additional file 4. [file 1678-9199-jvatitd-31-e20250015-s4.pdf]

J Venom Anim Toxins incl Trop Dis
https://doi.org/10.1590/1678-9199-JVATITD-2025-0015

Supplementary Material to "Cecal appendicitis as a rare manifestation of paracoccidioidomycosis: a case report and systematic review of the literature"

Additional file 4. Risk of bias of the studies included in the systematic review

Citation	Q1	Q2	Q3	Q4	Q5	Q6	Q7	Q8
Vianna* [10]	Yes	Yes	Yes	Yes	Yes	Yes	Yes	Yes
César et al. [11]	No	No	No	No	No	No	No	Yes
Barbosa et al. [12]	Yes	No	Yes	Yes	No	No	No	Yes
Bittencourt et al. [13]	Yes	Yes	Yes	Yes	Yes	Yes	Yes	Yes
Navas et al.** [14]	No	No	No	No	No	No	No	Yes
Muñoz-Urribarri et al. [15]	Yes	Yes	Yes	Yes	Yes	Yes	Yes	Yes
Gava et al. [16]	Yes	Yes	Yes	Yes	Unclear	No	Unclear	Yes
Luna-Vilchez et al. [17]	Yes	Yes	Yes	Yes	Yes	Yes	Yes	Yes
Sales et al.*** [18]	No	No	Yes	Yes	No	No	No	Yes
Marinho-Falcão et al. [19]	Yes	Yes	Yes	Yes	Yes	Yes	Yes	Yes
Present study****	Yes	Yes	Yes	Yes	Yes	Yes	Yes	Yes

Q1: Were the patient demographic characteristics clearly described?
Q2: Was the patient's history clearly described and presented in a timeline?
Q3: Was the current clinical condition of the patient on presentation clearly described?
Q4: Were the diagnostic tests or assessment methods and results clearly described?
Q5: Was the intervention(s) or treatment procedure(s) described clearly?
Q6: Was the postintervention clinical condition described?
Q7: Were adverse events (harmful) or unanticipated events identified and described?
Q8: Does the case report provide useful lessons?

*Thesis description. **Article not found, and data extracted from the abstract. ***Data extracted from conference poster. ****Data from this case report.
