# Supplementary material for: Cecal appendicitis as a rare manifestation of paracoccidioidomycosis: A case report and systematic review of the literature
Source: J Venom Anim Toxins Incl Trop Dis. 2025 Dec 8;31:e20250015. doi: 10.1590/1678-9199-JVATITD-2025-0015 (PMC12705073; doi:10.1590/1678-9199-JVATITD-2025-0015)
Supplement: Additional file 5. [file 1678-9199-jvatitd-31-e20250015-s5.pdf]

J Venom Anim Toxins incl Trop Dis

https://doi.org/10.1590/1678-9199-JVATITD-2025-0015

Supplementary Material to "Cecal appendicitis as a rare manifestation of paracoccidioidomycosis: a case report and systematic review of the literature"

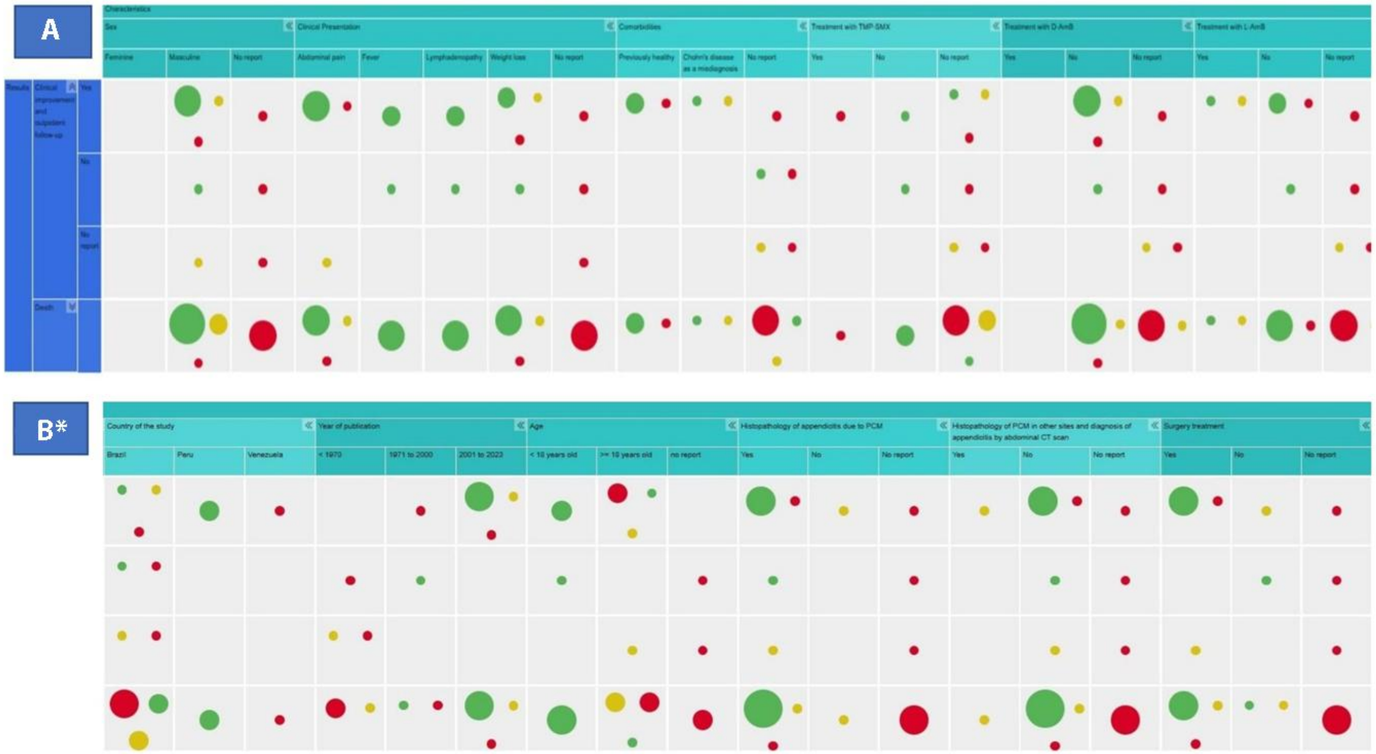

Additional file 5. Evidence map of case reports of cecal appendicitis caused by paracoccidioidomycosis.

*The evidence map was divided into two panels (A and B) to enhance readability for the readers. However, both figures represent the same continuous evidence map, presented in two parts for better visualization. The complete evidence map can be accessed at: https://eventos.matogrossodosul.fiocruz.br/mapas/apendicite_paracoc.html
